# The course of mental health after miscarriage and induced abortion: a longitudinal, five-year follow-up study

**DOI:** 10.1186/1741-7015-3-18

**Published:** 2005-12-12

**Authors:** Anne Nordal Broen, Torbjørn Moum, Anne Sejersted Bødtker, Øivind Ekeberg

**Affiliations:** 1 Department of Behavioral Sciences, Institute of Basic Sciences in Medicine, University of Oslo, P.O. Box 1111 Blindern, 0317 Oslo, Norway; 2 Department of Obstetrics and Gynecology, Buskerud Hospital, 3000 Drammen, Norway

## Abstract

**Background:**

Miscarriage and induced abortion are life events that can potentially cause mental distress. The objective of this study was to determine whether there are differences in the patterns of normalization of mental health scores after these two pregnancy termination events.

**Methods:**

Forty women who experienced miscarriages and 80 women who underwent abortions at the main hospital of Buskerud County in Norway were interviewed. All subjects completed the following questionnaires 10 days (T1), six months (T2), two years (T3) and five years (T4) after the pregnancy termination: Impact of Event Scale (IES), Quality of Life, Hospital Anxiety and Depression Scale (HADS), and another addressing their feelings about the pregnancy termination. Differential changes in mean scores were determined by analysis of covariance (ANCOVA) and inter-group differences were assessed by ordinary least squares methods.

**Results:**

Women who had experienced a miscarriage had more mental distress at 10 days and six months after the pregnancy termination than women who had undergone an abortion. However, women who had had a miscarriage exhibited significantly quicker improvement on IES scores for avoidance, grief, loss, guilt and anger throughout the observation period. Women who experienced induced abortion had significantly greater IES scores for avoidance and for the feelings of guilt, shame and relief than the miscarriage group at two and five years after the pregnancy termination (IES avoidance means: 3.2 vs 9.3 at T3, respectively, p < 0.001; 1.5 vs 8.3 at T4, respectively, p < 0.001). Compared with the general population, women who had undergone induced abortion had significantly higher HADS anxiety scores at all four interviews (p < 0.01 to p < 0.001), while women who had had a miscarriage had significantly higher anxiety scores only at T1 (p < 0.01).

**Conclusion:**

The course of psychological responses to miscarriage and abortion differed during the five-year period after the event. Women who had undergone an abortion exhibited higher scores during the follow-up period for some outcomes. The difference in the courses of responses may partly result from the different characteristics of the two pregnancy termination events.

## Background

Miscarriage is regarded as a difficult and distressing life event for a woman [1-3]. It can cause anxiety [4,5] and depression [6], and can also be experienced as a traumatic life event [7,8]. Results from research into the psychological implications of abortion are equivocal, and this has resulted in much debate, possibly because the theme is controversial on political, ethical and social grounds [9-12]. A recent review of post-1990 research articles [13] concluded that anxiety symptoms are the most common adverse response, and that our understanding of abortion as a potential trauma has increased. Recent studies have explored the traumatic aspects of abortion. One study reported that 1% of participants suffered from post-traumatic stress disorder (PTSD) two years after the event [12], and another reported that 10% of women were traumatized (according to a high Impact of Events Scale [IES] score) six months after the induced abortion [14]. In a previous study [15] in which the subjects were the same as those evaluated in this study, we found that 18.1% of women were classed as "cases" (> 19 points on one or both of the IES subscales) two years after an induced abortion.

Very few studies have compared the course of psychological responses after miscarriage with that after abortion. Induced abortion and miscarriage are similar life events in that women abort after a short term of pregnancy. However, the two life events differ in important respects. Miscarriage happens involuntarily and suddenly to women who were expecting to give birth a few months later, whereas abortion is a planned and known event. Women with unwanted pregnancies include those who are stable and content but have not finished their education or already have the number of children they desire. This category also includes women who have abortions because of financial difficulties, unstable relationships or chronic mental illness. An induced abortion is the result of a decision made after days or weeks of consideration, and the woman is mentally prepared when she arrives at the hospital. Nevertheless, the discovery of the pregnancy can be a shock, and the period prior to the abortion can be distressing. The process of deciding to have an abortion can be difficult, and the reason for electing to have an abortion can affect the psychological responses after the event [16]. Thus, the social, moral and psychological context of an induced abortion may be more complicated than that of a miscarriage, and may result in different psychological responses.

We hypothesized that women who undergo an induced abortion will have a more protracted course of mental disturbance than women who experience a miscarriage. Therefore, we compared the mental health outcomes of women who either experienced a miscarriage or underwent an induced abortion over a period of five years after the event using IES, Quality of Life, the Hospital Anxiety and Depression Scale (HADS), and feelings connected to the pregnancy termination.

## Methods

In Norway, induced abortion within the first 12 weeks of pregnancy became an unconditional legal right in 1978. Norway has approximately 4.6 million inhabitants; about 15,000 induced abortions and 8,000–10,000 miscarriages are treated in general hospitals per annum.

This study was approved by the Norwegian Regional Ethics Committee. Our study comprised 120 women between the ages of 18 and 45 years (80 of whom had had an induced abortion and 40 of whom had experienced a miscarriage), who were treated in the gynecology department of Buskerud Hospital between April 1998 and February 1999. Buskerud Hospital is the main hospital in Buskerud County and is situated in Drammen, a city of 55,000 citizens located 40 km west of Oslo, Norway. All women who had an induced abortion were less than 13 weeks pregnant, and no terminations were due to fetal anomalies. Of the women who experienced miscarriage, one was 21 weeks pregnant whereas the rest were less than 17 weeks pregnant. In our study, all surgery performed was completed under anesthesia, and the women left hospital a few hours after the procedure. The staff contacted the women shortly after the abortion while they were still in hospital. Those who agreed to participate in the study were then contacted by a female psychiatrist (ANB) employed in the psychiatry department of the hospital.

Two hundred and sixty-eight women were approached. Of these, 13 were excluded on the basis of defined exclusion criteria: (1) not Norwegian-speaking (n = 9); (2) mentally disabled or suffering from serious psychiatric illness (n = 3); and (3) pregnancy following rape (n = 1). Of the 255 women who were asked to participate, 120 (47%) agreed and were included (46% of the women who had had an induced abortion and 50% of those who had experienced a miscarriage). For women who had had an induced abortion, the response rate varied between 52% and 30%, depending on staff motivation and the person who asked the women to participate. When nurse G. asked the women, 52% agreed to participate in the study. For several years, this nurse had cared for women during the first hours after an induced abortion. She was genuinely interested in the project and had a positive attitude towards taking part in it. When other staff members asked the women, only 30% agreed to participate. The project leader (who was also the interviewer) was not well known to the staff, and some of the staff were skeptical about the study being carried out in their department. At the beginning of December 1998, when all but three of the women who had had an induced abortion were included, only half the women who had had a miscarriage were included. The project leader then had the opportunity to address the staff at a meeting that lasted for two hours. After this meeting, several staff members said that they were much more positive about the project than previously, and that they felt more comfortable about asking women to participate in the study. Before this meeting, the inclusion rate of women who had experienced a miscarriage was 36.5%; after the meeting it increased to 75%.

The mean ages of the women who had had an induced abortion and did or did not participate were 27.7 and 27.5 years, respectively (not statistically significant [n.s.]). The corresponding values for women who had had a miscarriage were 30.1 and 30.5 years (n.s.). We had no demographic information other than age for the women who did not participate in the study.

The women were interviewed 10 days (T1), six months (T2), two years (T3), and five years (T4) after the end of pregnancy. The interviews were semi-structured and included self-administered questionnaires. Of the 80 women who had had an induced abortion, 74 completed the interviews at T2, 72 at T3, and 70 at T4. Of the 40 women who had experienced a miscarriage, 40 completed the interviews at T2, 39 at T3, and 39 at T4. Thus, of the 120 women taking part in the project, 91% (43% of eligible women) completed the study.

At T1, all the women were asked if they felt that the time after the pregnancy termination had been difficult. Twelve women did not feel that it had been difficult (one of whom had had a miscarriage, 11 an induced abortion). All these women completed the study. Eleven women did not complete the study, one of whom experienced a miscarriage and 10 of whom had had an induced abortion. Of these, the woman who miscarried and seven of the women who had had induced abortions said that they wanted to discontinue their participation in the study because it was too difficult for them to answer questions about the pregnancy termination.

All interviews were conducted face-to-face by a female psychiatrist, except two at T3 (one by telephone, one by mail) and nine at T4 (eight by telephone, one by mail). The women's mental health before the pregnancy termination was measured by self-report and by diagnostic evaluation by the interviewer.

### Self-reported six-point scale assessment of the previous need for psychiatric help

1. No help ever required from health services.

2. No contact with or help from health services, but the woman felt that she had needed professional help on previous occasions.

3. The woman had consulted a general practitioner about psychological problems.

4. Previous contact with a private practitioner (psychiatrist or psychologist).

5. Previous treatment at a psychiatric outpatient clinic.

6. Previous inpatient treatment at a psychiatric clinic or at a clinic for substance abuse.

### Diagnostic evaluation

After the first interview, the women were assigned one or more ICD-10 (International Statistical Classification of Diseases, 10^th ^Revision) lifetime psychiatric diagnoses, if applicable. We devised a three-point scale, the Former Psychiatric Health Scale, based on a combination of the self-reporting assessment and the diagnostic evaluation:

1. Good. The woman rated herself as 1 or 2 and received no diagnosis from the psychiatrist.

2. Medium. The woman rated herself as 1 or 2, but was given a diagnosis by the psychiatrist.

3. Previous psychiatric problems. The woman rated herself as 3–6 and was given a diagnosis by the psychiatrist.

### Questionnaires

The following questionnaires were completed at all interviews.

#### Impact of Event Scale (IES)

The Impact of Event Scale [17] has been widely used as a measure of stress reactions after traumatic events. It has a two-factor structure: one measures intrusion (flashbacks, bad dreams, and strong feelings related to the traumatic event) and the other measures avoidance of thoughts and feelings related to the event. An evaluation of the scale after 20 years of use [18] reported that IES has been valuable for measuring stress reactions in a number of different populations. The type of event was shown to be a strong predictor of intrusive and avoidant symptoms after the traumatic event.

The IES version that we used contained 15 questions. Seven questions dealt with intrusion and eight dealt with avoidance. The women were asked to rate, on a scale from 0 to 5, their perceived level of specified symptoms during the previous week. The scale thus ranged from 0 to 35 for intrusion and from 0 to 40 for avoidance. Examples of questions on the intrusion scale are: "I have had bad dreams about the pregnancy termination" and "Things I have seen or heard suddenly reminded me of the pregnancy termination." Examples of questions on the avoidance scale are: "I know that I have many pent-up feelings about the pregnancy termination, but I have pushed them away", "I have tried not to talk about the pregnancy termination", and "I have not allowed myself to have thoughts about the pregnancy termination".

A recent review [19] showed that the IES is a reliable index of the degree of subjective distress associated with a particular trauma. A high score on the IES, especially on the intrusion scale, seems to be closely related to the presence of Acute Stress Disorder (ASD) or PTSD, as defined by the Diagnostic and Statistical Manual of Mental Disorders, Fourth Edition (DSM-IV). In our study, we did not use specific criteria for assigning these diagnoses but used the term "case", defined as a score of > 19 points on either of the two subscales, IES intrusion or IES avoidance, as is common practice [20,21].

#### Quality of life

The Quality of Life questionnaire that we used consisted of 12 items. The women were asked to choose between "never", "seldom", "sometimes", "often" or "all the time" to indicate the extent to which each of 12 statements applied to their lives during the previous two weeks. Examples of statements are: "I felt fit and strong", "I felt that life is worth living", and "I felt close to another person". The twelfth and last item was "When you think about how you are doing nowadays, are you mostly content with your life, or mostly discontent?" To this last item, the women were allowed to select from six different alternative answers. Thus, the total scores ranged from 12 to 61 points; the higher the score, the better the quality of life. Cronbach's alpha at the four interviews varied between 0.92 and 0.94. The questionnaire is a more comprehensive version of "Subjective Well-Being", which has been used in other studies in Norway [22-24]. The correlation between items in the version used in our study and "Subjective Well-Being" is 0.93. Normative values for this test are not available. However, an indication of normative values may be found in another study that used the same material to investigate hypertension screening [22]; the mean score for 60 women aged 25–45 years was 47.90 (SD = 7.60).

#### Hospital Anxiety and Depression Scale (HADS)

Zigmond and Snaith [25] introduced the HADS questionnaire in 1983. The questionnaire was shown to be valuable in detecting symptoms of anxiety and depression in a wide variety of patients [26]. It contains 14 questions, each rated from 0 to 3. Seven questions deal with anxiety during the previous week, and seven questions deal with depression during the previous week. The scores for anxiety and depression thus range from 0 to 21 points. For normative values, we used data from the "HUNT" (Helse Undersøkelse Nord Trøndelag) study, a large population study conducted in Norway from 1995 to 1997. This study was performed in the county of Northern Trøndelag (situated in the central part of Norway and containing about 3% of the population of Norway) [27]. Of all people aged between 20 and 89 years, 62,344 (67.7% of the total population) completed valid ratings of HADS. The data were kindly provided by Dr. Eystein Stordal. Women aged 30–35 years (n = 2,879) had the following mean scores: HADS anxiety = 4.6 ± 3.4, HADS depression = 2.6 ± 2.7. We used this age category for comparison because the women in our study had mean ages of 30.1 years (miscarriage) and 27.7 years (induced abortion) at T1, and 35.1 and 32.7 at T4, respectively.

#### Feelings associated with the abortion

Feelings after an induced abortion have been rated by other studies [9,12,28], which used Likert-type scales ranging from 1 (not at all) to 5 (extremely). We used a similar scale and measured the intensity of various feelings that the women experienced at the time of the interview when asked to think about the abortion. The participants were asked to rate their feelings of relief, grief, loss, guilt, shame and anger. For each feeling, they rated the intensity as either 1 (not at all), 2 (a little), 3 (a great deal), 4 (much) or 5 (very much).

### Statistics

The study was designed to detect "medium" effects when comparing the two abortion groups (defined as 0.5 by Cohen [29] and requiring sample sizes of approximately 70 individuals for each group when the alpha [type I] error level is set at 5% and the beta [type II] error level is set at 10%). After attrition, our study groups contained 70 (induced abortion) and 39 (miscarriage) participants at T4, yielding statistical power slightly above 70% for medium-sized effects and above 98% for "large (> 0.80) effects. This was considered satisfactory for our purposes.

Statistical associations between both pregnancy termination groups and other categorical independent variables were tested using the χ^2 ^test. Mean differences between pregnancy termination groups for continuous variables were tested by point biserial r/ANOVA (t-tests). The significance of changes in mean scores over time within each pregnancy termination group was tested with paired-sample t-tests. The significance of differential changes between groups was assessed by analysis of covariance (ANCOVA), using follow-up scores as the dependent variable, pregnancy termination group and categorical confounders as factors, and baseline scores for the outcomes as linear covariates (using the GLM procedure of SPSS). Effect sizes for changes are expressed as Cohen's d [29]. Partial product-moment correlations were computed between continuous outcome variables with linear controls for previous psychiatric health.

## Results

The characteristics of the women are shown in Table 1. There were statistically significant differences between the two pregnancy termination groups regarding their marital status, number of children and vocational activity. Therefore, these variables are possible confounders. As the outcomes of the study were related to mental outcomes, we also considered former psychiatric health (which was close to being significantly different between the two groups) to be a possible confounder.

**Table 1:** Characteristics at T1 of women participating in the study. Statistically significant differences between the two groups are shown.

	**Women with miscarriage, n = 40. ** **(Scored '1')**	**Women with induced abortion, n = 80. ** **(Scored '2')**	**Point biserial r/χ^2^**
**At T1 (10 days after the event)**	**Mean (95% CI)**	**Mean (95% CI)**	

**Age **(years)	30.1 (28.2–31.9)	27.7 (26.2–29.3)	r = -0.17, n.s.
**Length of pregnancy **(weeks)	10.5 (9.4–11.5)	9.6 (9.3–9.9)	r = -0.18, n.s.
**Number of previous induced abortions**	0.3 (0.1–0.5)	0.3 (0.2–0.4)	r = -0.02, n.s.
**Number of previous miscarriages**	0.4 (0.2–0.6)	0.4 (0.2–0.6)	r = 0.02, n.s.
**Number of children**	0.8 (0.5–1.0)	1.2 (0.9–1.4)	r = 0.19*
**Marital status**			χ^2 ^= 15.38***
Married	42.5%	21.3%	
Cohabitant	50.0%	37.5%	
Not married/cohabitant	7.5%	41.3%	
**Education**			χ^2 ^= 5.42, n.s..
Comprehensive school up to 16 years of age	10.0%	15.0%	
Comprehensive school up to 19 years of age	15.0%	31.3%	
Vocational education	47.5%	31.3%	
University education	27.5%	22.5%	
**Vocational activity**			χ^2 ^= 10.34*
Still in education	2.5%	21.3%	
Regular employment	75.0%	50.0%	
Temporary employment	5.0%	11.3%	
Working at home	10.0%	8.8%	
Other	7.5%	8.8%	
**Religious faith**			χ^2 ^= 5.05, n.s.
Christian, the faith is of minor importance	80.0%	71.3%	
Christian, the faith is of great importance	12.5%	6.3%	
Agnostic or humanistic ethicist	5.0%	17.5%	
Muslim or other	2.5%	5.0%	
**Former psychiatric health**			χ^2 ^= 3.63, n.s.
Good	65.0%	47.5%	
Medium	15.0%	17.5%	
Previous psychiatric problems	20.0%	35.0%	

χ^2 ^(Pearson's χ^2^) = for pregnancy termination group by nominal variable

r (Pearson's r, t-test) = for pregnancy termination type by continuous variables

*p < 0.05, **p < 0.01, ***p < 0.001.

Table 2 shows the mean scores from all mental health questionnaires for each pregnancy termination group. The results of Table 2 are illustrated in the figures, below.

**Table 2:** Mean outcome scores and standard deviations (SD) for both pregnancy termination groups.

	**T1 **Ten days after pregnancy termination Mean ± (SD)		**T2 **Six months after pregnancy termination Mean ± (SD)		**T3 **Two years after pregnancy termination Mean ± (SD)		**T4 **Five years after pregnancy termination Mean ± (SD)	
**Outcome**	**Mis-carriage** ** n = 40**	**Induced abortion** ** n = 80**	**Mis-carriage** ** n = 40**	**Induced abortion** ** n = 74**	**Mis-carriage** ** n = 39**	**Induced abortion** ** n = 72**	**Mis-carriage** ** n = 39**	**Induced abortion** ** n = 70**

**IES intrusion**	17.6** ± (9.5)	11.9 ± (9.3)	10.6 ± (8.7)	8.0 ± (8.6)	4.9 ± (5.3)	5.1 ± (5.6)	3.7 ± (4.5)	3.6 ± (5.9)
**IES avoidance**	7.0 ± (6.1)	11.1** ± (7.9)	5.9 ± (6.4)	9.7* ± (8.6)	3.2 ± (4.6)	9.3*** ± (9.4)	1.5 ± (3.6)	8.3*** ± (10.1)
**Quality of Life**	42.2 ± (9.0)	41.4 ± (9.1)	45.6 ± (7.9)	43.6 ± (8.7)	47.7 ± (7.8)	45.3 ± (7.9)	47.4 ± (6.5)	45.9 ± (8.2)
**HADS anxiety**	6.1 ± (4.1)	6.6 ± (4.6)	5.5 ± (4.1)	6.8 ± (5.0)	5.6 ± (4.1)	6.0 ± (4.7)	5.2 ± (4.2)	5.9 ± (4.6)
**HADS depression**	4.5 ± (4.2)	3.9 ± (4.0)	3.0 ± (3.3)	3.3 ± (3.7)	2.3 ± (3.2)	2.6 ± (3.7)	2.0 ± (2.6)	2.7 ± (3.2)
**Feelings, rated 1–5:**								
**Relief**	1.3 ± (0.7)	2.8*** ± (1.4)	1.3 ± (0.6)	2.6*** ± (1.4)	1.3 ± (0.8)	2.7*** ± (1.3)	1.4 ± (0.9)	2.7*** ± (1.4)
**Grief**	3.7*** ± (1.5)	2.4 ± (1.4)	3.2*** ± (1.3)	2.2 ± (1.2)	2.4* ± (1.2)	1.9 ± (1.0)	1.8 ± (0.9)	1.8 ± (1.0)
**Loss**	3.6*** ± (1.5)	2.2 ± (1.4)	3.4*** ± (1.4)	2.2 ± (1.3)	2.5 ± (1.2)	2.2 ± (1.3)	2.0 ± (1.1)	1.9 ± (1.1)
**Guilt**	1.9 ± (1.2)	2.1 ± (1.4)	1.5 ± (0.9)	2.1** ± (1.2)	1.2 ± (0.7)	1.9** ± (1.0)	1.1 ± (0.2)	2.0*** ± (1.1)
**Shame**	1.1 ± (0.3)	1.8*** ± (1.3)	1.1 ± (0.4)	1.9*** ± (1.3)	1.1 ± (0.4)	1.6** ± (1.0)	1.0 ± (0.0)	1.6** ± (1.0)
**Anger**	2.2 ± (1.3)	1.8 ± (1.3)	2.0 ± (1.1)	1.9 ± (1.3)	1.5 ± (1.0)	1.8 ± (1.1)	1.3 ± (0.7)	1.5 ± (1.0)

Statistically significant differences between pregnancy termination groups at all time points: *p < 0.05, **p < 0.01, ***p < 0.001

At T1, women who had experienced a miscarriage had significantly higher IES intrusion scores than those of women who had experienced an induced abortion (17.6 vs 11.9, respectively; p < 0.01), but this was not the case at any subsequent time-point.

Women who had had an induced abortion had IES avoidance scores significantly higher than those of women who had had a miscarriage at T1 (11.1 vs 7.0, respectively; p < 0.01), T2 (9.7 vs 5.9, respectively; p < 0.05), T3 (9.3 vs 3.2, respectively; p < 0.001), and T4 (8.3 vs 1.5, respectively; p < 0.001).

**Figure 3:**
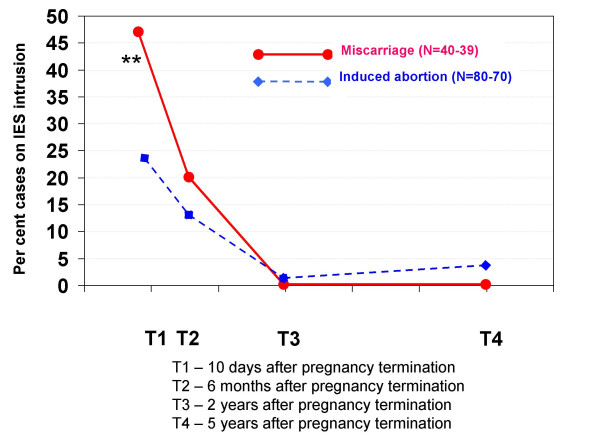
**Percentage of cases according to IES intrusion in each pregnancy termination group at all four interviews**. IES intrusion is a psychological trauma test that measures the women's extent of intrusive thoughts, feelings and flashbacks about the pregnancy termination event. A high score (> 19 points) on this scale indicates a "case". Statistically significant differences between the groups: * p < 0.05, ** p < 0.01, *** p < 0.001.

**Figure 4:**
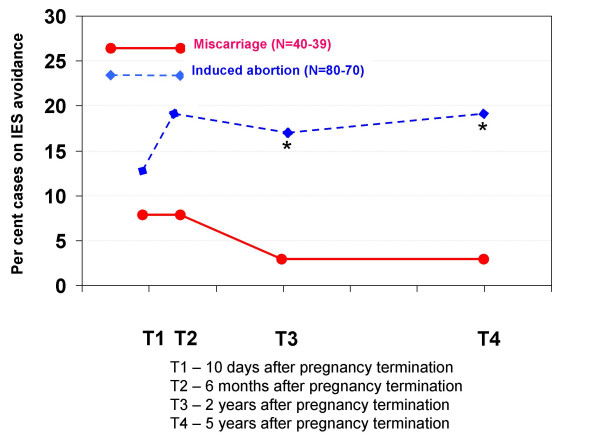
**Percentage of cases according to IES avoidance in each pregnancy termination group at all four interviews**. IES avoidance is a psychological trauma test that measures how much the women avoid thinking, talking or feeling anything about the pregnancy termination event. A high score (> 19 points) on this scale indicates a "case". Statistically significant differences between the groups: * p < 0.05, ** p < 0.01, *** p < 0.001.

The cases on the IES (> 19 points on each subscale) are shown in Figures 3 and 4. Figure 3 shows the percentage of IES intrusion cases in each pregnancy termination group during the five years after the event.

The group of women who had experienced a miscarriage initially had a high percentage of intrusion cases, but there were no cases at later interviews: T1 = 47.5%, T2 = 20.0%, T3 = 0%, T4 = 0%. The corresponding values for women with induced abortions were: T1 = 23.8%, T2 = 13.5%, T3 = 1.4%, T4 = 4.3%.

Figure 4 shows the percentages of IES avoidance cases in each pregnancy termination group during the five years after the event.

Women who had experienced a miscarriage had a relatively low initial percentage of avoidance cases, which decreased at subsequent interviews: T1 = 7.5%, T2 = 7.5%, T3 = 2.6%, T4 = 2.6%. Among women who had had an induced abortion, the number of avoidance cases was consistently elevated at all four interviews (T1 = 12.5%, T2 = 18.9%, T3 = 16.7%, T4 = 18.6%). The total proportion of women who were cases according to one or both IES subscales were T1 = 47.5%, T2 = 22.5%, T3 = 2.6%, T4 = 2.6% for women who had experienced miscarriage, and T1 = 30.0%, T2 = 25.7%, T3 = 18.1%, T4 = 20.0% for women who had had an induced abortion.

**Figure 5:**
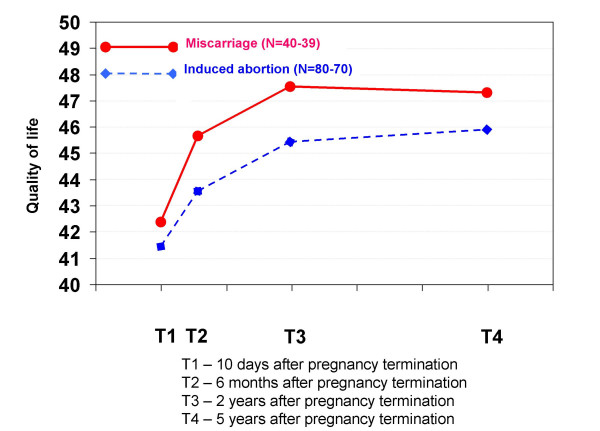
**Mean Quality of Life scores in each pregnancy termination group at all four interviews**. The Quality of Life test measures how satisfied subjects are with their own lives. The higher the score, the better the quality of life. Statistically significant differences between groups: * p < 0.05, ** p < 0.01, *** p < 0.001.

Figure 5 shows that Quality of Life scores were not significantly different between the two groups at any time and that they improved in both groups during the study period.

**Figure 6:**
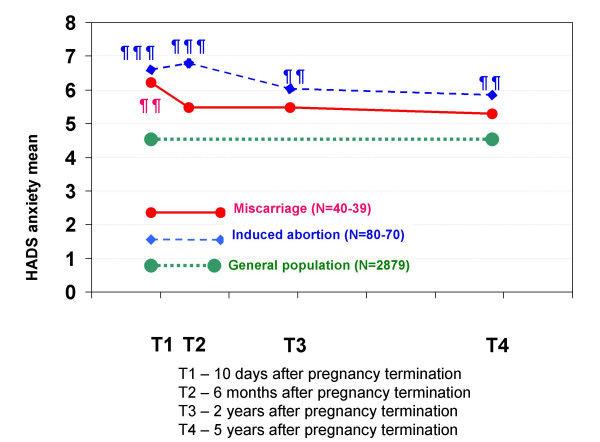
**Mean HADS anxiety scores in each pregnancy termination group at all four interviews**. The mean anxiety scores for the two pregnancy termination groups and the mean anxiety scores for women in a general population sample in Norway (HUNT) are shown. There were no statistically significant differences between the two pregnancy termination groups. Statistically significant differences between the scores of each pregnancy termination group and those of the general population sample (of women aged 30–35 years, n = 2,879): ¶ p < 0.05, ¶¶ p < 0.01, ¶¶¶ p < 0.001.

**Figure 7:**
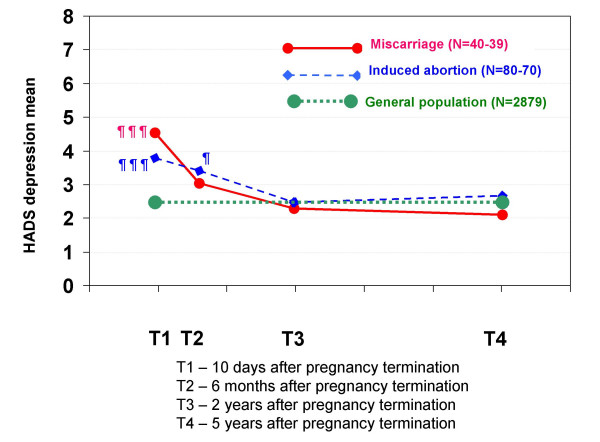
**Mean HADS depression scores in each pregnancy termination group at all four interviews**. The mean depression scores of the two pregnancy termination groups and the mean depression scores of women in a general population sample in Norway (HUNT) are shown. There were no statistically significant differences between the two pregnancy termination groups. Statistically significant differences between the scores of each pregnancy termination group and those of the general population sample (of women aged 30–35 years, n = 2,879): ¶ p < 0.05, ¶¶ p < 0.01, ¶¶¶ p < 0.001.

The HADS scores (Figures 6 and 7) were not significantly different between the two groups. However, compared with the mean HADS scores of the general population, women who had experienced a miscarriage had significantly higher anxiety (p < 0.01) and depression (p < 0.001) scores at T1, but not at the later interviews. Compared with the general population, women who had had an induced abortion had significantly higher anxiety scores at all four interviews (p < 0.001 to p < 0.01), and significantly higher depression scores at T1 (p < 0.001) and T2 (p < 0.05).

Regarding feelings related to the pregnancy termination, women who had experienced a miscarriage had significantly more grief at T1, T2 and T3 and significantly more feelings of loss at T1 and T2 than the other group. Women who had had an induced abortion had significantly more relief at all interviews than women who had had a miscarriage, but this variable did not increase during the five-year period. They also had significantly more guilt at T2, T3 and T4, and more shame at all interviews.

When scores for the mental health outcomes of the two groups (Table 2) were compared with those of controls for possible confounders (marital status, number of children, vocational activity and former psychiatric health), differences in IES avoidance at T1 and T2 were no longer statistically significant. Furthermore, the difference between groups was reduced for IES avoidance at T3 (p < 0.01), IES avoidance at T4 (p < 0.01), guilt at T2 (p < 0.05), shame at T1 (p < 0.01), shame at T2 (p < 0.01) and shame at T3 (p < 0.05). On the other hand, the difference between groups was more statistically significant for IES intrusion at T1 (p < 0.001).

Table 3 shows the changes in mental health scores for all women throughout the study period after all four possible confounders are controlled for.

**Table 3:** Changes in mental health outcomes between T1 and T4 in each pregnancy termination group. Cohen's d estimates the changes in outcome variables in the two pregnancy termination groups. Within-group analysis was performed using paired t-tests and between-group analysis was performed using ANCOVA (estimated with controls for all four possible confounders, i.e. marital status, number of children, vocational activity and former psychiatric health).

Outcome at corresponding interviews:	Change in the **miscarriage **group from T1 to T4: Cohen's d	Change in the **induced abortion **group from T1 to T4: Cohen's d	Differential change between groups from T1–T4: Exact p-value
**IES intrusion**	1.92***	0.85***	p = 0.096
**IES avoidance**	1.20***	0.21**	**p = 0.003**
**Quality of life**	-0.69***	-0.40**	p = 0.628
**HADS anxiety**	0.10	0.02	p = 0.999
**HADS depression**	0.80**	0.30*	p = 0.314
**Feelings, rated 1–5:**			
**Relief**	-0.50	0.07	p = 0.081
**Grief**	1.54***	0.41***	**p = 0.015**
**Loss**	1.22***	0.08	**p = 0.007**
**Guilt**	0.93***	0.00	**p < 0.001**
**Shame**	0.47	0.17	p = 0.088
**Anger**	0.86***	0.34**	**p = 0.027**

T1 = 10 days after pregnancy termination

T4 = five years after pregnancy termination

Significant change from T1 to T4 (in each pregnancy termination group) by paired t-test: *p < 0.05, **p < 0.01, ***p < 0.001.

In both groups, the outcomes changed significantly from T1 to T4 for IES intrusion, IES avoidance, Quality of Life, and HADS depression, grief and anger, but not for HADS anxiety, relief or shame. Women who had experienced a miscarriage also had significantly ameliorated feelings of loss and guilt over the period of observation, but this was not true of women who had had an induced abortion.

The pattern of changes in mental health scores over the study period differed between the two pregnancy termination groups. The changes in levels of IES avoidance, grief, loss, guilt and anger from T1 to T4 were significantly greater for women who had experienced a miscarriage than for those who had had an induced abortion. In contrast, none of the outcome levels changed significantly more for women who had had an induced abortion than for women who had experienced a miscarriage.

Because the results of the study revealed that elevated scores for IES avoidance persisted for two and five years after the event for women who had had an induced abortion, partial correlations were estimated between IES avoidance at T3 and T4 and mental health outcomes, HADS, Quality of Life, and feelings at the corresponding interviews. There were statistically significant correlations (from p < 0.05 to p < 0.001) with all the negative health outcomes (except HADS depression at T3 and HADS depression and less relief at T4). The analyses controlled for former psychiatric health.

## Discussion

Our hypothesis that there would be a protracted course of psychological responses in women who had had an induced abortion was supported by some of the responses measured in this study. Women who had had a miscarriage experienced the sudden termination of pregnancy as a traumatic and sad life event. Almost half the women were "cases" according to their score on the IES at T1, and they scored high on feelings of grief and loss at T1 and T2. During the five-year follow-up period, they improved more rapidly according to their scores on the IES avoidance, grief, loss, guilt and anger than women who had had an induced abortion.

In both groups, the HADS anxiety scores were high relative to those of the general population. This was especially true for the induced abortion group, for which the mean anxiety scores were statistically higher than those of the general population at all four interviews. Anxiety after induced abortions has been the topic of other studies. Higher rates of subsequent generalized anxiety were recently reported among aborting women than among women who had carried an unintended pregnancy to term [30]. The authors stated that no causal relationship between pregnancy outcome and anxiety could be determined. Despite this, they remarked that their findings of more generalized anxiety among aborting women were consistent with the results of other studies, which also noted that anxiety was a possible negative effect of induced abortion [13,31]. In our study, aborting women had somewhat higher (although non-significant) levels of anxiety than miscarrying women. This finding may imply that induced abortion resulted in more anxiety than miscarriage. However, the mental health of aborting women was poorer (almost statistically significantly) than that of miscarrying women prior to the pregnancy termination event. Therefore, we cannot infer that induced abortion caused the elevated anxiety of the induced abortion group relative to that of the miscarriage group.

The induced abortion group had significantly higher anxiety scores than the general population at all interviews, whereas the miscarriage group only had significantly higher anxiety scores at T1. This indicates that either the mental health of the aborting women was different from that of the general population before and after the abortion event or that the induced abortion led to anxiety that persisted for several years after the abortion. An appropriate experimental design is required to answer this question.

Other mental health outcomes, such as depression, trauma responses, quality of life and feelings, may likewise be poorer for women in the induced abortion group because of their mental health status before the abortion.

In our study, anxiety was not significantly reduced from T1 to T4 in either group, and the rate of change from T1 to T4 was not significantly different between women who had experienced a miscarriage and those who had had an induced abortion. Recent review articles indicate that anxiety is more important after miscarriage and induced abortion than has been recognized to date [4,13].

Women who had had an induced abortion experienced a more protracted course of IES avoidance. Their IES avoidance scores remained high and were almost unchanged throughout the five years, whereas their IES intrusion scores fell with time. In the miscarriage group, both scores on IES subscales decreased simultaneously, as is common with trauma responses. An explanation for the unusual and divergent courses of the IES scores in the induced abortion group is not obvious, but may result from the characteristics of the abortion event.

Our findings of high IES avoidance scores in the induced abortion group are in agreement with results from a study in which trauma responses after abortion were examined in American and Russian women [32]. Many women had avoidance symptoms related to the induced abortion several years after the event (for American women, the mean was about 10 years after the event; for Russian women, the mean was about six years after the event). Among the American women, 50% avoided thinking or talking about the abortion, compared with 19% of the Russian women. About 25% of the American women had difficulties being near babies, compared with 4% of the Russian women. Of the American women, 36% had three or more avoidance symptoms, compared with 3% of the Russian women. This study indicates that cultural differences influence psychological responses to induced abortion. The results of our study imply that post-abortion avoidance responses among Norwegian women are more similar to those of American women than to those of Russian women.

In our study, 30% of the women who had had an induced abortion were IES cases at T1 according to one or both IES subscales. Five years after the abortion, 20% were still cases. Most of these cases resulted from high IES avoidance scores. Classification as a "case" according to the IES indicates that the person suffers from some degree of mental distress, although it does not mean she is suffering from PTSD. However, the IES is a psychological trauma test and is recommended for screening possible PTSD sufferers [19]. For those women who had had an induced abortion, the partial correlation tests showed that high IES avoidance scores at T3 and T4 correlated with most other concurrent negative mental health scores.

The elevated scores for guilt, shame and IES avoidance for women who had had an induced abortion may require more attention. Several recent studies have focused on the relationship between guilt, shame and PTSD [33-35]. One article states that "the affects of shame and guilt in particular can be very disabling, in so far as they ... affect the experience of the self and social behaviour, contribute to later psychopathology, effect help-seeking, and impede emotional processing of the event." [36]. In our previous article [15], we found that feelings of guilt and shame 10 days after a pregnancy termination predicted high IES avoidance scores two years later (a statistical interaction effect showed that this tendency was even more important for women who had had an induced abortion). It is possible that feelings of guilt and shame associated with the induced abortion contribute to a slower improvement in mental health.

Women who had had an induced abortion had high scores for relief throughout the study period. This indicates that their situation shortly before the abortion was experienced as very difficult and stressful. Other studies confirm this observation of relief after an induced abortion [9,12,37].

### Limitations and strengths of the study

The introduction of the new parameter "former psychiatric health" may be a limitation of the study because the validity and reliability of this scale has not been tested. However, the assessment was based on observations by an experienced psychiatrist and on reports by the women on rather robust aspects of mental health, such as whether they had been treated for psychiatric problems previously. In most of the analyses we controlled for former psychiatric health, but we cannot exclude possible bias due to (unmeasured) differences in mental health between the two pregnancy termination groups before the event.

Another limitation of the study is the lack of control for all prior and subsequent pregnancy outcomes. We did apply controls for a few of these possible confounding factors, but did not detect any statistically significant effect of prior miscarriages or induced abortions on scores for IES or feelings at T1, T2 or T3. Nor did we observe any statistically significant effect of childbirth events between T1 and T4 on the HADS anxiety and depression scores at T4. This finding should be tested in a study with a larger sample and extended to include the effects of subsequent miscarriages and abortions.

The low participation rate (47%) is another limitation of the study. Regarding the nature and direction of possible selection bias, a former study has shown that those who do not participate in studies such as this have more problems than those who do participate [38]. Another report [39] demonstrated selection bias in a study of how women experienced induced abortion one year after the event. One third of the women did not want to participate; these women were overrepresented on certain sociodemographic factors (young, unmarried, low educational status) that have been shown to be associated with increased vulnerability and morbidity. However, we cannot know what those women who did not participate in this study would have scored on the psychological tests and this constitutes a limitation.

Another limitation arises from the selection of the participants. As described in the Methods section, there was an overrepresentation of women who coped well with the termination among those who completed the study. This was particularly evident for women who had had an induced abortion. Therefore, the results at T2, T3 and T4 may have been biased towards overly favorable mental health outcomes.

The high follow-up rate (109 of 120 women [91%] completed all four interviews) and the long duration of the follow-up period strengthen the study.

## Conclusion

The responses of women in the miscarriage group were similar to those expected after a traumatic and sad life event. However, the women in the induced abortion group had more atypical responses. This may be because the mental health of the aborting women was somewhat poorer than that of the miscarrying women before the pregnancy termination event. The more complex nature of the induced abortion event may also account for differences in the course of psychological responses between the two groups.

Women in both groups should be given information about common psychological responses to pregnancy termination, and follow-up talks with health personnel should be offered to women most affected by the event.

## Competing interests

The author(s) declare that they have no competing interests.

## Authors' contributions

ANB contributed to the design of the study, conducted all the interviews, participated in the analysis of the data, and drafted and completed the manuscript. TM conducted the data analysis, participated in drafting the manuscript, and revised it critically for intellectual content. ASB contributed to the design of the study and the acquisition of data, and revised the manuscript critically for intellectual content. ØE contributed to the design of the study, the interpretation of data, and revised the manuscript critically for intellectual content. All the authors have read and approved the final manuscript.

**Figure 1:**
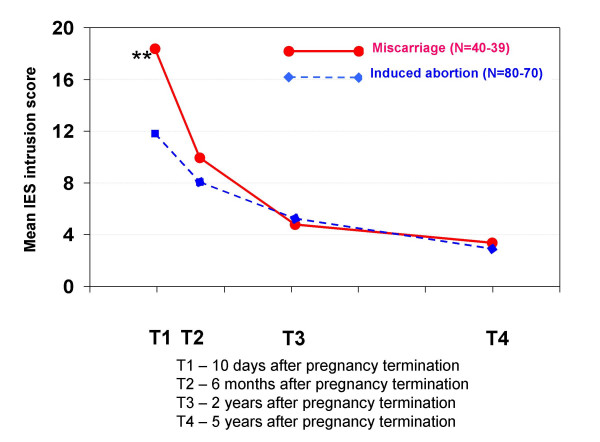
**Mean IES intrusion scores in each pregnancy termination group at all four interviews**. IES intrusion is a psychological trauma test that measures the extent of intrusive thoughts, feelings and flashbacks about the pregnancy termination event. Statistically significant differences between the groups: * p < 0.05, ** p < 0.01, *** p < 0.001.

**Figure 2:**
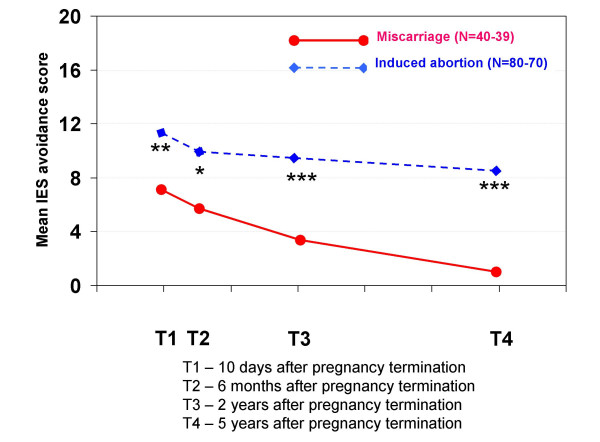
**Mean IES avoidance scores in each pregnancy termination group at all four interviews**. IES avoidance is a psychological trauma test that measures how much women avoid thinking, talking or feeling anything about the pregnancy termination event. Statistically significant differences between the groups: * p < 0.05, ** p < 0.01, *** p < 0.001.

**Figure 8:**
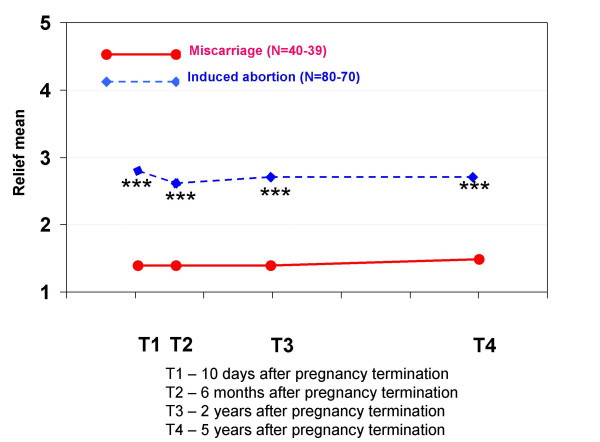
**Mean scores for feeling relief in each pregnancy termination group at all four interviews**. At each interview, the women were asked to indicate how much relief they felt when thinking about the pregnancy termination. The scores were: 1 (not at all), 2 (a little), 3 (a great deal), 4 (much) and 5 (very much). Statistically significant differences between the groups: * p < 0.05, ** p < 0.01, *** p < 0.001.

**Figure 9:**
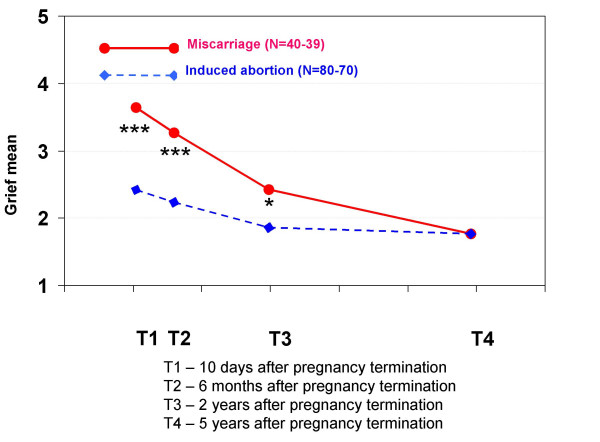
**Mean scores for feeling grief in each pregnancy termination group at all four interviews**. At each interview, the women were asked to indicate how much grief they felt when thinking about the pregnancy termination. The scores were: 1 (not at all), 2 (a little), 3 (a great deal), 4 (much) and 5 (very much). Statistically significant differences between the groups: * p < 0.05, ** p < 0.01, *** p < 0.001.

**Figure 10:**
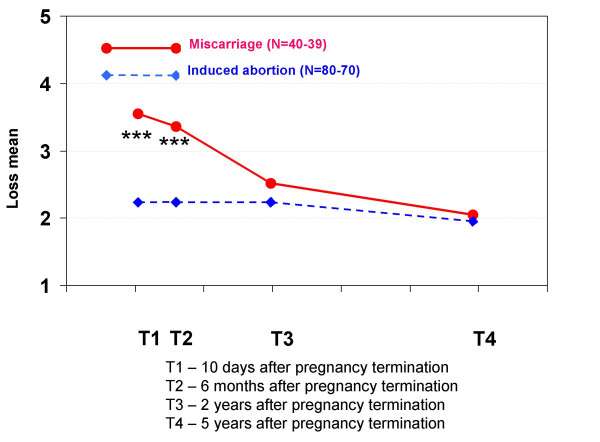
**Mean scores for feeling loss in each pregnancy termination group at all four interviews**. At each interview, the women were asked to indicate how much loss they felt when thinking about the pregnancy termination. The scores were: 1 (not at all), 2 (a little), 3 (a great deal), 4 (much) and 5 (very much). Statistically significant differences between the groups: * p < 0.05, ** p < 0.01, *** p < 0.001.

**Figure 11:**
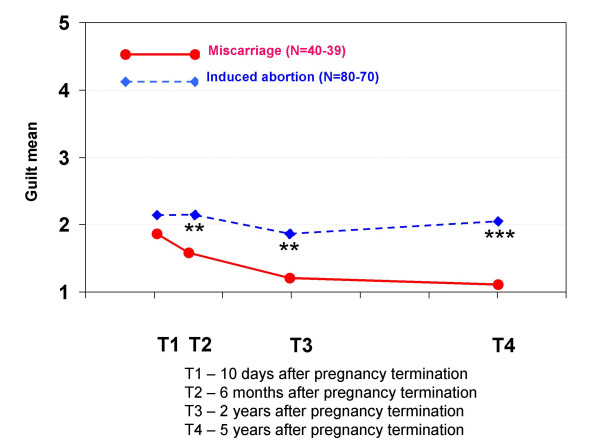
**Mean scores for feeling guilt in each pregnancy termination group at all four interviews**. At each interview, the women were asked to indicate how much guilt they felt when thinking about the pregnancy termination. The scores were: 1 (not at all), 2 (a little), 3 (a great deal), 4 (much) and 5 (very much). Statistically significant differences between the groups: * p < 0.05, ** p < 0.01, *** p < 0.001.

**Figure 12:**
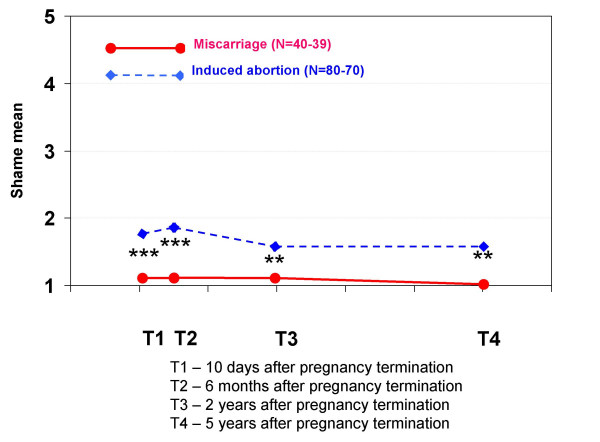
**Mean scores for feeling shame in each pregnancy termination group at all four interviews**. At each interview, the women were asked to indicate how much shame they felt when thinking about the pregnancy termination. The scores were: 1 (not at all), 2 (a little), 3 (a great deal), 4 (much) and 5 (very much). Statistically significant differences between the groups: * p < 0.05, ** p < 0.01, *** p < 0.001.

**Figure 13:**
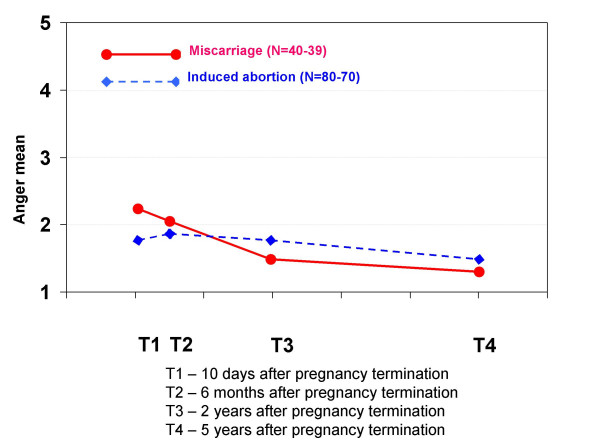
**Mean scores for feeling anger in each pregnancy termination group at all four interviews**. At each interview, the women were asked to indicate how much anger they felt when thinking about the pregnancy termination. The scores were: 1 (not at all), 2 (a little), 3 (a great deal), 4 (much) and 5 (very much). Statistically significant differences between the groups: * p < 0.05, ** p < 0.01, *** p < 0.001.

## Pre-publication history

The pre-publication history for this paper can be accessed here:


